# Opsoclonus-myoclonus syndrome associated with multiple system atrophy

**DOI:** 10.1186/s40673-014-0015-6

**Published:** 2014-11-01

**Authors:** Kazumasa Shindo, Akiko Onohara, Takanori Hata, Fumikazu Kobayashi, Kaori Nagasaka, Takamura Nagasaka, Yoshihisa Takiyama

**Affiliations:** Department of Neurology, University of Yamanashi Hospital, 1110 Tamaho, Yamanashi, 409-3898 Japan

**Keywords:** Opsoclonus-myoclonus syndrome, Multiple system atrophy, Ocular symptom, Cerebellar vermis, Pontine reticular formation

## Abstract

Opsoclonus-myoclonus syndrome (OMS) is well known as a paraneoplastic syndrome or as a parainfectious neurologic complication. However, OMS associated with a neurodegenerative disorder has not been described previously. A 48-year-old woman had been diagnosed as multiple system atrophy-parkinsonian type (MSA-P) based on the findings of dopamine non-responsive parkinsonism with autonomic failure and typical findings on magnetic resonance imaging 5 years ago. She exhibited recurrent asynchronous and arrhythmic myoclonic movements of the upper limbs and abdomen with a very short duration, and involuntary eye movements, which were repetitive, rapid, random, multidirectional, conjugate saccades of irregular amplitude and frequency at rest. Based on hematological and radiological findings, the diagnosis was advanced MSA-P associated with OMS. As far as we are aware, there have not been any previous reports of such a case.

**Electronic supplementary material:**

The online version of this article (doi:10.1186/s40673-014-0015-6) contains supplementary material, which is available to authorized users.

## Background

Opsoclonus-myoclonus syndrome (OMS) is well known as a paraneoplastic syndrome, especially in children with neuroblastoma, or as a parainfectious neurologic complication of diseases such as HIV infection [1–4]. In previous reports, other causes that have been documented include multiple sclerosis, cerebrovascular disease, sarcoidosis, pregnancy, autoimmune disorders, and adverse reactions to several drugs. However, OMS associated with a neurodegenerative disorder has not been described previously [2,3,5–7]. Here we report a patient with advanced multiple system atrophy-parkinsonian type (MSA-P) who developed OMS. As far as we are aware, there have not been any previous reports of such a case.

## Case report

A 48-year-old woman had presented 7 years ago with left hand tremor, gait disturbance related to bradykinesia of all four limbs, and postural instability. Decreased sweating during summer and recurrent syncope after standing due to an excessive fall of blood pressure had been noted 5 years ago, and MSA-P had been diagnosed based on the findings of dopamine non-responsive parkinsonism combined with autonomic failure and abnormal linear signal intensities in the putamen bilaterally on magnetic resonance imaging. Her bradykinesia and rigidity gradually increased, resulting in inability to walk and stand unassisted, while oral intake became impaired by dysphagia due to pseudobulbar palsy 1 year ago. In April 2012, she was admitted to our hospital because of frequent episodes of dizziness even in the supine position and transient episodes of involuntary twitching of her upper limbs for 3 months. There was a history of hyperthyroidism when she was 41 years old, which had been treated and was well controlled. Her family history was unremarkable.

On admission, supine blood pressure was 136/74 mmHg and it decreased to 82/46 mmHg with head-up tilt of 45 degrees. Neurological examination revealed mild dementia, a very small voice, severe dysphagia, moderate rigidity of the neck and all limbs, severe bradykinesia, exaggerated deep tendon reflexes, positive pathological reflexes in the lower limbs, diffuse muscle atrophy due to disuse, and autonomic symptoms/signs such as systemic anhydrosis, orthostatic hypotension, constipation, and dysuria. She exhibited recurrent asynchronous and arrhythmic myoclonic movements of the upper limbs and abdomen with a very short duration, which occurred at rest and were also induced by sudden sound, touch or painful stimuli. Very mild cerebellar ataxia was found in her upper limbs, but palatal tremor was not present. Ophthalmic examination (Additional file 1) demonstrated involuntary eye movements, which were repetitive, rapid, random, multidirectional, conjugate saccades of irregular amplitude and frequency at rest. These movements continued whether her eyes were open or closed and became more exaggerated when she attempted to follow the finger of an examiner.

**Figure 1:**
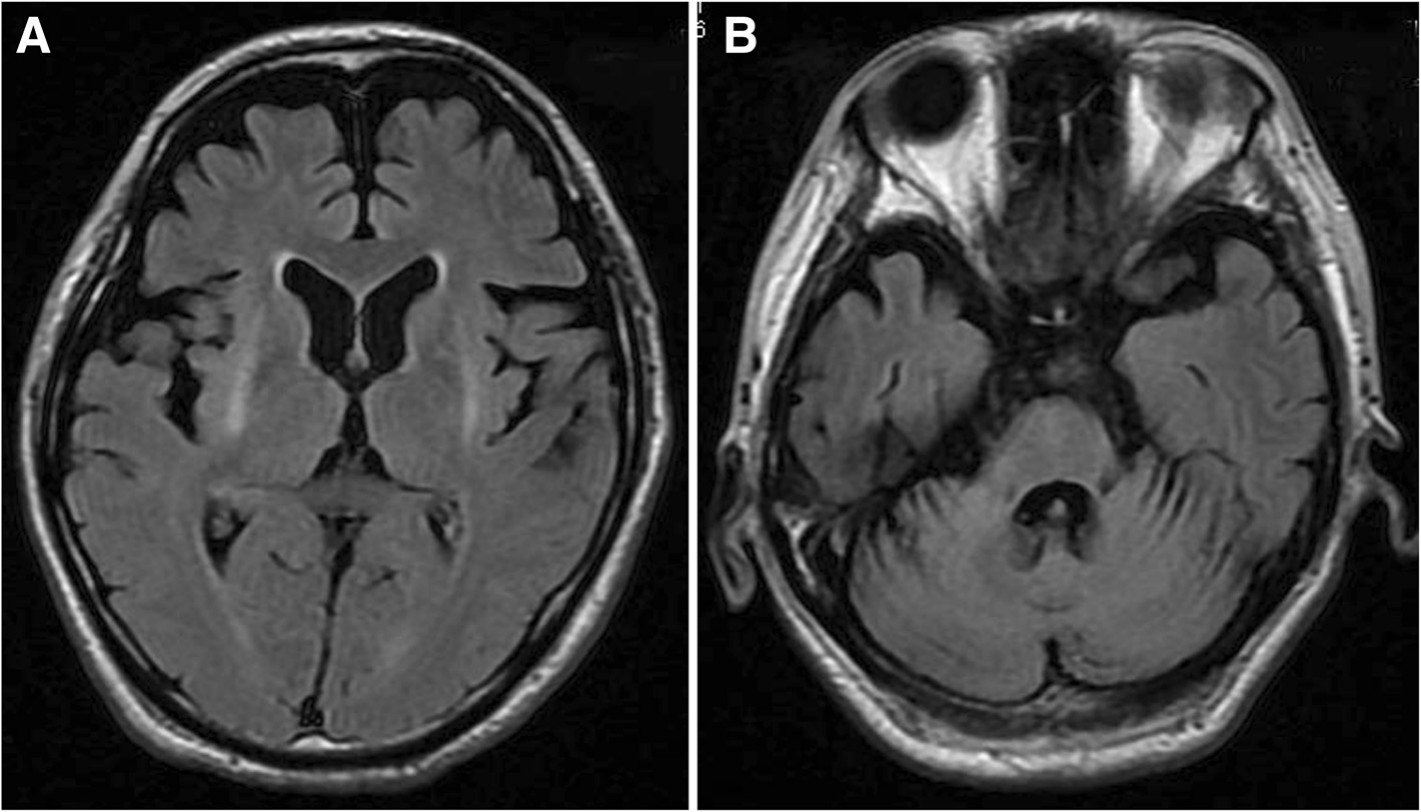
**Fluid attenuated inversion recovery images on brain MRI. A**, **B**. Fluid attenuated inversion recovery images on MRI demonstrated linear high signal intensity of putamen bilaterally and mild cerebellar and pontine atrophy.

**Figure 2:**
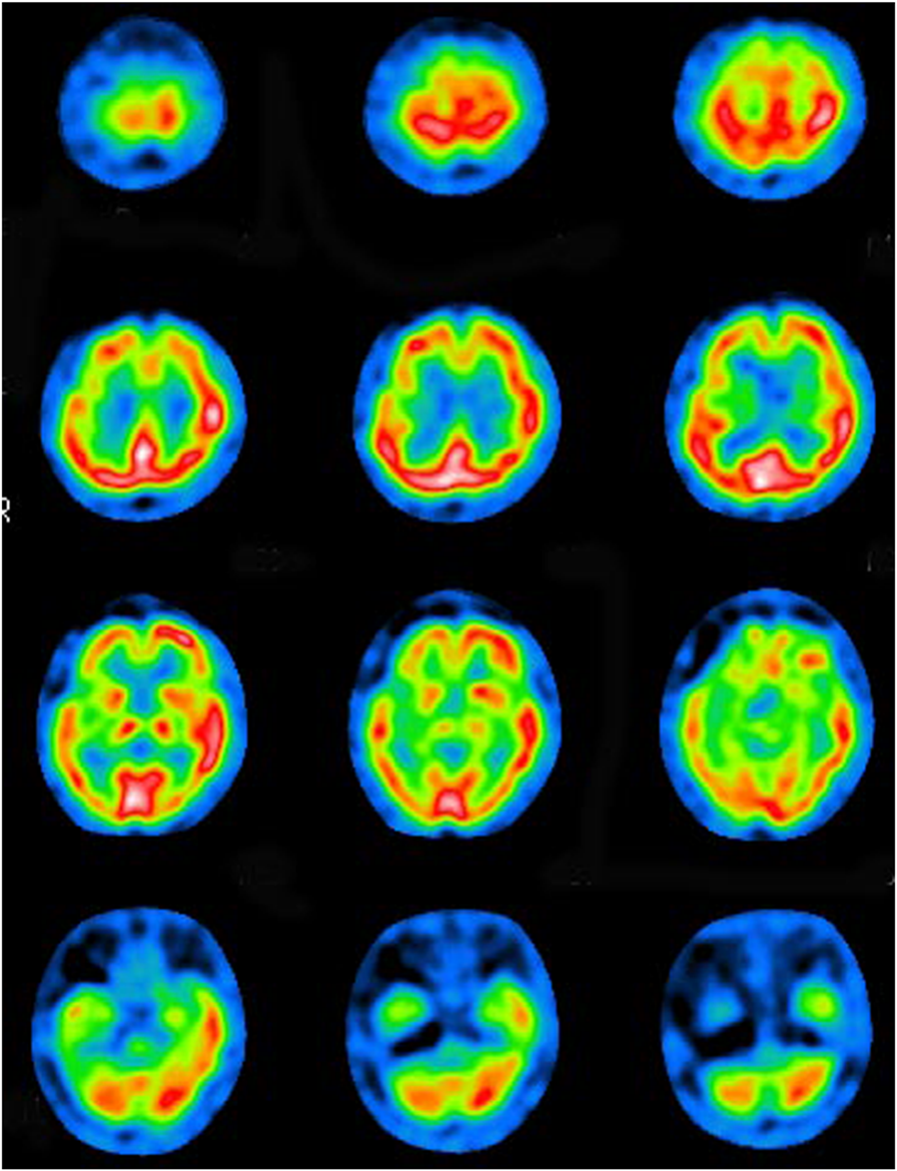
**Single photon emission computed tomography imaging revealed obvious hypoperfusion of the right frontal and temporal cortices, basal ganglia and brainstem, while perfusion of right cerebellar cortex was mildly impaired.**

Routine blood tests and examination of cerebrospinal fluid (CSF) showed no abnormalities. There were no significant changes in serum or CSF antibodies for HIV, herpes simplex virus, Epstein-Barr virus, cytomegalovirus, varicella-zoster virus, Japanese encephalitis A and B virus, and JC virus. Assays for anti-nuclear antibody, anti-double-stranded DNA antibody, anti-nuclear cytoplasmic antibody, anti-thyroid antibodies, and anti-phospholipid antibody were all negative. CSF examination with a paraneoplastic panel (anti-Hu, anti-Ri, anti-Yo, anti-MaTa, anti-VGKC, amphyphysin antibodies, anti-gliadin antibodies) was also unrevealing. During myoclonic jerk movements of the upper limbs, electroencephalography only showed generalized slowing of background activity without epileptic discharges. The giant sensory evoked potential was not observed on examination of somatosensory evoked potentials. Full-body computed tomography (CT) demonstrated mild aspiration pneumonia in the right lower lobe, but no evidence of either a primary or metastatic tumor was found. Magnetic resonance imaging (MRI) revealed linear high signal intensities in the putamen bilaterally, as well as mild cerebellar and pontine atrophy on T2-weighted and FLAIR images (Figure 1). Single photon emission computed tomography (SPECT) with Tc-99 m-ECD revealed obvious hypoperfusion of the cerebrum, basal ganglia, and brainstem, while perfusion of the cerebellum only showed a slight decrease (Figure 2).

Based on these findings, the diagnosis was advanced MSA-P associated with OMS. Treatment of her involuntary movements was performed with clonazepam, valproic acid, gabapentin, levetiracetam, trihexyphenidyl, levodopa, and baclofen. Although subjective dizziness at rest and jerky limbs myoclonus almost resolved, a mild degree of opsoclonus persisted as of July 2014.

## Discussion

In adults, OMS typically presents as a paraneoplastic syndrome that is usually related to lung cancer or other malignancies [1–3]. Our patient showed no elevation of tumor markers and was negative for paraneoplastic antibodies. CT of the thorax and breasts was also unremarkable, although endoscopic evaluation of the gastrointestinal tract was not performed due to severe akinesia and dysphagia. Moreover, opsoclonus still persisted at two years after the onset of OMS, while nothing suggestive of malignancies was found by physical examination and imaging studies. Thus, paraneoplastic syndrome was considered to be excluded as a differential diagnosis. Other possible etiologies, such as parainfectious syndrome, direct infection of the brain, cerebrovascular disease, and autoimmune disease were excluded from the clinical course, antibody studies, normal CSF findings, and brain MRI findings. We therefore considered that this patient had OMS accompanied by advanced MSA.

Patients with MSA have been reported to show various ocular symptoms such as saccadic pursuit, blurred vision, nystagmus, anisocoria, Horner’s syndrome, facial dystonia, and limitation of upward gaze, downward gaze, and horizontal gaze [8,9]. However, opsoclonus has not been previously reported in a patient with MSA or in other parkinsonian syndromes. On the other hand, jerky tremor or distal myoclonus, with an irregular myoclonic postural or action tremor of the distal limbs, has been reported in 3 ~ 31% of MSA patients and it is one of the features supporting diagnosis of this disease [8–10].

Although the pathophysiological mechanism of OMS remains unclear, lesions of two neuroanatomical structures have been suggested to be responsible based on histological and biochemical studies. One possibility is a cerebellar lesion interrupting normal vermian inhibition of the fastigial nucleus, which regulates vestibular nuclear tone to burst neurons [1–3]. The other possibility is a lesion of the caudal pontine reticular formation affecting the function of pause neurons that inhibit the burst neurons that drive saccades [2,5,6]. It is also speculated that reticular formation lesions could lead to hyperexcitability of burst neurons. Cerebellar lesions involving the vermis and pontine lesions affecting the reticular formation have often been demonstrated by previous neuropathological studies [11,12], so opsoclonus might be expected in patients with MSA. It is speculated that opsoclonus was observed in the present case due to lesions of the brainstem and basal ganglia along with comparative preservation of cerebellar function based on the physical and radiological findings up to the advanced stage, because cerebellar hyperperfusion or activation has been detected by SPECT or fMRI in previous investigations of OMS as a paraneoplastic syndrome [4,13,14].

Since opsoclonus is a characteristic feature in ocular involuntary movements, detection of opsoclonus in addition to jerky myoclonic tremor might be useful as one of the supporting features for diagnosis of MSA. To conclude the causative relationship between OMS and MSA, statistical analysis of the frequency of opsoclonus in a large population of MSA patients may be important.

## Conclusion

Opsoclonus-myoclonus syndrome associated with a neurodegenerative disorder has not been described previously. In a 48-year-old woman diagnosed as multiple system atrophy, recurrent arrhythmic myoclonic movements of the upper limbs and abdomen, and involuntary eye movements, which were repetitive, rapid, random, multidirectional, conjugate saccades of irregular amplitude and frequency were observed at rest. Based on hematological and radiological findings, the diagnosis was advanced multiple system atrophy associated with opsoclonus-myoclonus syndrome. As far as we are aware, there have not been any previous reports of such a case.

## Consent statement

Written informed consent was obtained from the patient for publication of this case report and any accompanying images. A copy of the written consent is available for review by Editor-in-Chief of this journal.

## Additional file

Additional file 1:
**Involuntary eye movements of repetitive, rapid, random, multidirectional, conjugate saccades with irregular amplitude and frequency was demonstrated at rest.** These movements continued with eye open and closed and were exaggerated when she gazed fingers of examiners.

## Abbreviations

MSA: Multiple system atrophy
OMS: Opsoclonus-myoclonus syndrome

## References

[1] Sotirchos ES, Dorsey ER, Tan IL, Zee DS (2011). Opsoclonus-myoclonus syndrome and exaggerated startle response associated with small-cell lung cancer. Mov Disord.

[2] Wong AMF, Musallam S, Tomlinson RD, Shannon P, Sharpe JA (2001). Opsoclonus in three dimensions: oculographic, neuropathologic and modelling correlates. J Neurol Sci.

[3] Klaas JP, Ahlskog JE, Pittock SJ, Matsumoto JY, Aksamit A, Bartleson JD, Kumar R, McEvoy KF, McKeon A (2012). Adult- onset opsoclonus-myoclonus syndrome. Arch Neurol.

[4] Van Toorn R, Rabie H, Warwick JM (2005). Opsoclonus-myoclonus in an HIV-infected child on antiretroviral therapy-possible immune reconstitution inflammatory syndrome. Eur J Pediatr Neurol.

[5] Hattori T, Hirayama K, Imai T, Yamada T, Kojima S (1988). Pontine lesion in opsoclonus-myoclonus syndrome shown by MRI. J Neurol Neurosurg Psychiatry.

[6] Baets J, Pals P, Bergmans B, Foncke E, Smets K, Hauman H, Vanderwegen L, Cras P (2006). Opsoclonus-myoclonus syndrome: a clinicopathological confrontation. Acta Neurol Belg.

[7] Boland T, Strause J, Hu M, Santamaria D, Liang TW, Kremens D, Sergott R, Moussouttas M (2012). Posterior reversible encephalopathy syndrome presenting as opsoclonus-myoclonus. Neuro-Ophthalmology.

[8] Wenning GK, Shlomo B, Magalhaes M, Daniel SE, Quinn NP (1994). Clinical features and natural history of multiple system atrophy. An analysis of 100 cases. Brain.

[9] Wenning GK, Tison F, Shlomo B, Daniel SE, Quinn NP (1997). Multiple system atrophy: A review of 203 pathologically proven cases. Mov Disord.

[10] Gilman S, Wenning GK, Low PA, Brooks DJ, Mathias CJ, Trojanowski JQ, Wood NW, Colosimo C, Dürr A, Fowler CJ, Kaufmann H, Klockgether T, Lees A, Poewe W, Quinn N, Revesz T, Robertson D, Sandroni P, Seppi K, Vidailhet M (2008). Second consensus statement on the diagnosis of multiple system atrophy. Neurology.

[11] Kume A, Takahashi A, Hashizume Y, Asai J (1991). A histometrical and comparative study on Purkinje cell loss and olivary nucleus cell loss in multiple system atrophy. J Neurol Sci.

[12] Braak H, Rub U, Del Tredici K (2003). Involvement of precerebellar nuclei in multiple system atrophy. Neuropathol Appl Neurobiol.

[13] Oguro K, Kobayashi J, Aiba H, Hojo H (1997). Opsoclonus-myoclonus syndrome with abnormal single photon emission computed tomography imaging. Pediatr Neurol.

[14] Helmchen C, Rambold H, Sprenger A, Erdmann C, Binkofski F (2003). Cerebellar activation in opsoclonus. An fMRI study. Neurology.

